# Federated high order tensor fusion for privacy preserving multimodal social media analysis

**DOI:** 10.1371/journal.pone.0344980

**Published:** 2026-05-06

**Authors:** Li Wan, Bin Zhang

**Affiliations:** School of Information Engineering, Hunan University of Science and Engineering, Hunan, China; Chengdu University of Traditional Chinese Medicine Wenjiang Campus: Chengdu University of Traditional Chinese Medicine, CHINA

## Abstract

The rapid evolution of social networks has positioned multimodal content, including text, images, and audio, as a pivotal medium for self-expression and public sentiment analysis. However, existing multimodal fusion methods are often limited by privacy risks, parameter redundancy, and insufficient exploitation of intermodal correlations. To overcome these challenges, this study introduces a novel federated learning framework that integrates high-order tensor-based multimodal data fusion with privacy-aware decentralized training by keeping raw data local. It leverages tensor Tucker decomposition to capture complex spatial and semantic relationships between modalities, enhancing fusion accuracy while supporting user privacy through local data retention. Experimental results on the separate TREC2017 Precision Medicine Track Scientific Abstracts dataset and on the CMU-MOSI multimodal sentiment benchmark demonstrate that the proposed algorithm outperforms existing methods. The TREC2017 experiments validate the framework’s performance in text-dominant conditions (higher Mean Average Precision, MAP)), while the CMU-MOSI experiments confirm the effectiveness of the high-order tensor fusion in modeling intermodal correlations for multimodal tasks. Furthermore, our framework demonstrates adaptive learning capabilities, efficiently processing diverse multimodal data types without expanding redundant model parameters. This research opens new avenues for privacy-aware multimodal data fusion in social media, offering a robust solution for monitoring and managing online public opinion while supporting user privacy through local data retention.

## 1. Introduction

The rapid evolution of social media has positioned multimodal content, including text, images, and audio, as a pivotal medium for public sentiment analysis. Social media platforms such as forums, blogs, QQ, microblogs, and WeChat have emerged as significant avenues for internet users to express their viewpoints and emotions [1], offering a vast pool of online information resources that play a critical role in users’ work, study, and daily activities [2]. Online public opinion, amplified through these platforms, reflects the perspectives, attitudes, and emotions of internet users [3], and is characterized by diversity, autonomy, interactivity, and suddenness. Consequently, enhancing the monitoring and management of social media opinion dissemination has become a key focus for both scholars and industry regulators.

Despite the richness of multimodal data, existing fusion approaches face several critical limitations. Centralized data aggregation poses significant risks to user privacy, as sensitive information stored on servers becomes vulnerable to breaches [4]. Moreover, models with redundant parameters are often impractical for deployment on edge devices due to limited computational and storage resources [5]. Additionally, correlations between different modalities remain underutilized, leading to suboptimal performance in sentiment analysis and public opinion monitoring [6]. The proposed method employs tensor Tucker decomposition, which represents multimodal data as high-order tensors to model complex spatial and semantic interactions, facilitating a more comprehensive exploitation of intermodal correlations compared to traditional techniques such as independent modality processing or simple feature concatenation. Here, intermodal correlations refer to the complex spatial (e.g., alignment between visual and textual elements) and semantic (e.g., complementary emotional cues across modalities) relationships between data types, rather than purely statistical measures such as correlation coefficients.

Data fusion is essential to leverage the complementary information derived from different modalities, thereby enabling a more comprehensive understanding of user sentiment and behavior. For example, text data may offer explicit opinions, whereas images and audio can reveal implicit emotions and contextual nuances. Through the integration of these modalities, a holistic representation of user expressions can be achieved. this is particularly evident in recent social science methodologies that treat social media images as primary data to capture behavioral insights that text alone might miss [7] . This comprehensive perspective is vital for ensuring accurate sentiment analysis and effective public opinion monitoring. The development of deep learning techniques has brought new opportunities in the field of multimodal data fusion [8]. Deep learning plays a significant role in natural language processing, image processing, and speech recognition [9–13]. Galassi et al. [14] modeled the processed text by considering the linkage relationships between contextual words through a three-layer neural network to derive a vector representation of words, overcoming the shortcomings of statistical language models. Rezaeenour et al. [15] used recurrent neural networks (RNNs) to build text-processing models. RNNs can learn the contextual information of text well, but they face issues such as gradient disappearance. Yang et al. [16] proposed a long short-term memory (LSTM) model. The introduction of a gate mechanism in the LSTM compensates for the shortcomings such as gradient disappearance or gradient explosion encountered in RNNs during model training. Abdallah et al. [17] proposed a network model that combines the attention mechanism with convolutional neural networks (CNN) to process social media text data.

The proliferation of multimodal social media content offers unprecedented opportunities for analysing public sentiment, but centralized data fusion risks user privacy, redundant model parameters hinder deployment on edge devices, and intermodal correlations remain underutilized. To address these challenges, this work proposes a federated learning framework that fuses high-order tensor representations of multimodal data while preserving privacy. Our method enables decentralized training across edge nodes, adaptively captures spatial and semantic relationships between modalities, and significantly reduces communication costs. By leveraging tensor decomposition techniques, we compress the model parameters, making it feasible for deployment on resource-constrained edge devices. This approach not only enhances the efficiency of multimodal data fusion but also ensures that user privacy is maintained throughout the process. The current empirical validation is carried out on publicly available benchmark datasets (TREC2017 and CMU-MOSI) in a simulated federated setting. Extension to large-scale, real-world social media corpora (e.g., Twitter/Weibo/Reddit) with full distributed deployment is left as future work.

After fusing the data using our proposed high-order tensor decomposition, the next steps include (1) applying the federated learning framework to perform decentralized model training across distributed edge devices, ensuring privacy preservation, and (2) using the fused, privacy-protected multimodal representations for downstream tasks such as sentiment classification, trend detection, and public opinion monitoring. This full pipeline, from feature extraction and multimodal fusion to federated learning and application, is designed to address the dual challenges of leveraging heterogeneous social media data while respecting user privacy and computational constraints.

This research aims to develop a framework for privacy-preserving, decentralized learning. It utilizes multimodal social media data, including text, images, and audio. The goal is to improve the accuracy and robustness of public opinion and sentiment analysis. Current methods face disadvantages such as centralized data aggregation risking privacy, redundant parameters limiting edge deployment, and underutilized intermodal correlations. Our framework integrates federated learning with high-order tensor-based fusion, enabling decentralized training, efficient parameter reduction, and enhanced correlation capture through tensor Tucker decomposition. This tensor-based approach ensures that the fused representation fully utilizes the complementary nature of multimodal data, leading to improved performance in sentiment analysis and public opinion monitoring.

The main contributions of this study are as follow:

(1) Customized feature extraction: Adaptation of existing frameworks to handle the unique characteristics of social media data, ensuring relevance to the target application.
(2) Tensor-Based Feature Fusion: A novel fusion mechanism that leverages tensor Tucker decomposition to capture complex relationships between modalities, proven effective through extensive experiments.
(3) Privacy-preserving federated learning: A decentralized training approach that preserves user privacy, reduces communication costs, and enables efficient deployment on edge devices.
(4) Experimental validation: The proposed method was evaluated on two complementary benchmarks. The TREC2017 Precision Medicine Track dataset is used to assess the method’s behavior in a text-dominant retrieval setting, while the CMU-MOSI dataset is employed to evaluate the framework’s multimodal fusion capability. Performance was measured using MAP and standard multimodal classification metrics to ensure comparability with prior work.

## 2. State of the art

### 2.1. Multimodal data fusion

As the Internet and social media continue to advance, users increasingly employ various modalities, including text, images, expressions, videos, and more, to express their emotions. Consequently, multimodal data has emerged as a crucial data source for sentiment analysis. By conducting joint feature analysis on multimodal data, it becomes possible to uncover the intricate relationships between different modalities and enhance the accuracy of feature recognition. The fusion of multimodal data information can be achieved through feature layer fusion, decision layer fusion, and consistency regression fusion techniques.

(1) Feature layer fusion method

The feature layer fusion, also known as early fusion, involves the extraction of features from each modality individually. Subsequently, the extracted features from different modalities are combined, and the resulting fused features are fed into a classifier for classification [18]. In the work by Yang et al. [19], the authors initially extracted text features and color features, as well as texture features from images. They then employed a feature mapping approach to map these features from different modalities into the same feature space. Huang et al [20] used a tree-structured LSTM model for feature analysis of text and images in order to better map between words and image regions in the text. Experimental comparisons were made with other fusion models and the model showed superior results. Liang et al [21] used the SCCA method to fuse expression features and speech features, and the classifier used *K* nearest neighbors to achieve dual-modal emotion recognition for facial expression and speech. For multimodal data analysis with feature layer fusion, although the information between the two modalities is considered comprehensively, the problem of differences between the data and the problem of data redundancy and dimensional disaster are not considered.

(2) Decision-level fusion method

Decision-level fusion is late fusion. It first extracts the features of each modality, and each modality constructs its own classification model separately; finally, the resulting classification results are fused for decision-making using fusion rules. Recent advances in decision-level fusion have demonstrated improved performance through sophisticated weighting strategies. For instance, Yang et al. [22] proposed an adaptive meta-learning framework for dynamic weight assignment in multimodal sentiment analysis, which automatically adjusts the contribution of each modality based on context and data quality. Similarly, Dui et al. [23] developed a hierarchical attention mechanism for decision-level fusion that captures both intra-modality and inter-modality dependencies, significantly enhancing fusion accuracy in social media applications. Furthermore, Chen et al. [24] introduced a contrastive learning approach for decision-level fusion that improves model robustness against missing modalities, a common challenge in real-world social media environments. Although the decision-level fusion of multimodal data requires a high classification model for each modality, its fusion method is flexible and plays a role between two modalities at the same time.

(3) Consistent regression fusion method

Consistency regression fusion combines different modalities in a hybrid approach to multimodal data fusion. One approach includes developing a bidirectional bimodal fusion network that can fuse and separate pairs of modal representations. This network leverages these bimodal pairs as input and employs the gating mechanism within the Transformer architecture to optimize the final output. Another method introduces a deep modular collaborative attention network designed to combine keywords from questions with key targets in images, facilitating tasks related to visual question-answering. Additionally, researchers constructed an RNN model with two attention layers for multimodal sentiment analysis. The first attention layer fuses and reduces the dimensionality of the data, while the second attention layer enhances the underlying architecture to capture salient aspects of contextual information within the discourse.

### 2.2. Federated learning

In 2016, Google’s McMahan et al [25] introduced the concept of federated learning. Its main idea is to improve the efficiency of machine learning algorithms using distributed computing while protecting personal data privacy. In federated learning, edge nodes secure user privacy to a certain extent by exchanging local model parameters instead of raw data. In addition, federated learning is able to provide efficient deep learning for multiple participants or compute nodes, and is therefore widely used in a variety of fields, such as healthcare, finance, social media, etc.

Federated learning is a machine learning method in which several edge nodes storing data are able to perform collaborative training under the premise of data security and data privacy. It works as follows: the central parameter server first selects edge nodes that meet the conditions to participate in training and sends the global model to each edge nodes, which replaces the local model with the global model. Edge nodes use local data for model training and upload the trained model parameters to the server. Finally the server aggregates the parameters from different edge nodes and updates them to get a new global model.

Depending on the direction of the application, scholars have categorized federated learning into “cross-device” and “cross-silo” federated learning. Federated learning was initially proposed with an emphasis on applications in mobile and edge devices, i.e., cross-device federated learning. The main characteristics of its application scenarios include: (1) non-independent and identically distributed user data; (2) unbalanced user data; (3) large-scale distribution; and (4) limited communication resources. According to the type of data silos and the way of data division, federated learning can be categorized into (1) horizontal federated learning, (2) vertical federated learning, and (3) federated migration learning.

Federated learning has many advantages. First, compared with cloud computing models, federated learning only sends updated model parameters for aggregation, which greatly reduces the cost of data communication and improves the utilization of network bandwidth. Second, the user’s raw data does not need to be sent to the cloud, which avoids the possibility of leaking the user’s privacy when the data is uploaded to the link. Furthermore, the model training of federated learning can be trained and decided in real-time on edge nodes or end devices, and the latency will be greatly improved compared to the decision making in the cloud.

Within social media, federated learning enables local model training on users’ devices or nodes. This decentralized training approach not only ensures privacy preservation but also captures the nuances of individual user behaviors. Each user essentially becomes a player in this distributed game, contributing unique strategies based on their interactions within the social media space.

The application of federated learning in social media information dissemination is multi-faceted. Firstly, it addresses the privacy concerns associated with centralized models by design, as individual user data remains on their respective devices, never centralized on a server. This not only aligns with contemporary privacy regulations but also engenders trust among users who are increasingly concerned about the security of their personal information. Secondly, federated learning enables personalized and context-aware models in dynamic social media environments with diverse content and evolving user preferences. Federated learning, with its decentralized learning approach, adapts to these dynamics by incorporating individual user behaviors and preferences into the model. This results in more accurate and personalized content recommendations, enhancing the overall user experience. Furthermore, federated learning facilitates robustness and fairness in social media algorithms. The decentralized nature of federated learning mitigates biases that may arise in centralized models by ensuring that the training data is diverse and representative of the entire user base. This is crucial in social media platforms, where diverse voices and perspectives should be accounted for to avoid reinforcing existing biases.

The field of federated learning has evolved rapidly to address challenges such as statistical heterogeneity and communication efficiency. A notable study by Abhijit et al. [26] introduced a personalized federated learning algorithm using hypernetworks to generate edge nodes-specific models, which is highly relevant for social media applications with non-IID data. Furthermore, some scholars provide a comprehensive overview of advances in privacy-enhancing technologies integrated with federated learning, highlighting the ongoing effort to balance model performance with rigorous privacy guarantees.

In summary, federated learning is a powerful paradigm in machine learning, providing a decentralized and privacy-preserving approach to collaborative model training. Its applications span various domains, with particular significance in the dynamic landscape of social media information dissemination.

## 3. Methodology

### 3.1. Federated learning system model

**Fig 1:**
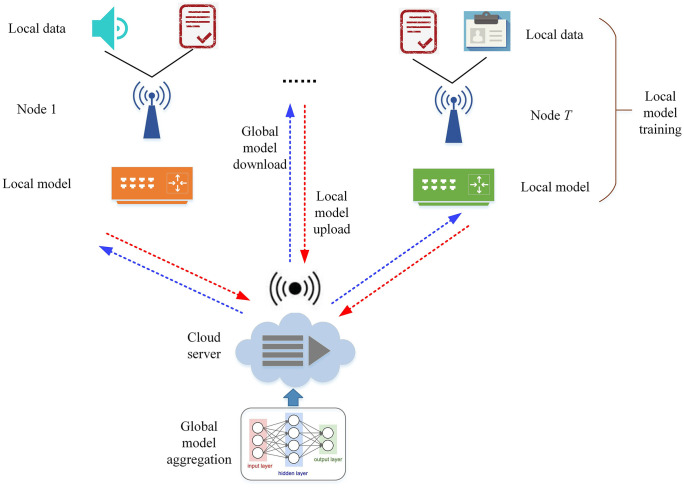
Architecture of the federated learning system. The system consists of edge nodes, IoT devices, and a cloud server. The arrows indicate the flow of model parameters between edge nodes and the server during federated training, illustrating the decentralized and privacy-preserving learning process.

To enable the learning of latent features from multiple users’ multimodal data while supporting user privacy through local data retention, this study proposes the integration of federated learning into edge computing. Fig 1 illustrates the fundamental architecture of the system. This setup facilitates secure and efficient communication between the edge nodes and the central cloud server, ensuring the privacy of users’ data during the learning process.

The system model presented in Fig 1 demonstrates the application of federated learning. Through iterative interactions with the cloud server, the model gradually improves its performance. With the proposed framework, federated learning enables collaborative training of a shared model using local data.

The proposed federated learning framework is highly scalable, supporting numerous heterogeneous devices with diverse computational capacities. By leveraging edge computing, the system distributes the computational load across multiple edge nodes, ensuring efficient resource utilization. The decentralized nature of the framework allows it to seamlessly integrate new devices into the network without requiring significant modifications to the existing infrastructure. This scalability is particularly advantageous in social media environments, where the number of users and devices can vary significantly over time.

### 3.2. Algorithm design overview

**Fig 2:**
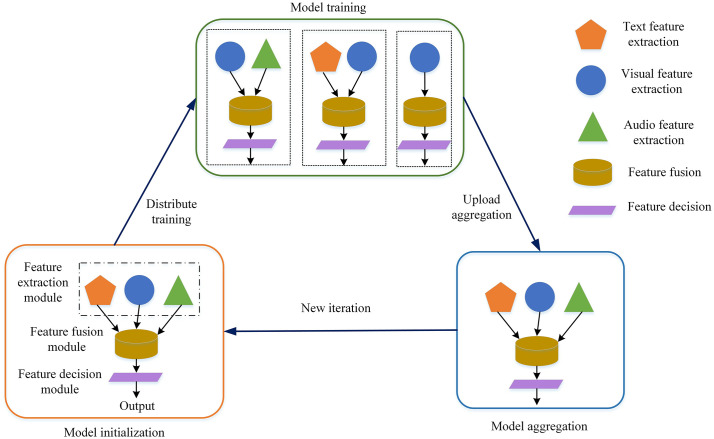
General framework of the proposed algorithm. The diagram illustrates the three core modules: feature extraction, feature fusion, and feature decision. The process flow from local model training on edge nodes to global model aggregation on the central server is depicted.

As depicted in Fig 2, the multimodal data fusion algorithm based on federated learning in this paper is comprised of three main modules: feature extraction, feature fusion, and feature decision. During the model training phase, each edge node receives the model from the central control node and selects the appropriate feature extraction module based on the local dataset’s structure (e.g., modality-specific sub-networks for text, audio, or visual data, as detailed in Section 3.3.1). Training at the edge nodes continues until the local training rounds exceed the predetermined threshold (e.g., 5 local epochs per communication round as in our experiments), which balances local computation with global communication efficiency to reduce overhead in federated learning [25,27]. In the model aggregation phase, the average aggregation algorithm is employed to combine the feature fusion and feature decision modules (i.e., by computing weighted averages of their parameters across nodes to update the global model, as detailed in Section 3.4; this guides convergence by integrating diverse local updates without relying on high/low parameter values as indicators).

The proposed federated learning framework for high-order tensor-based multimodal data fusion involves several key components, each with its own computational complexity. Here is a detailed breakdown:

1) Feature extraction:

Audio and Visual Data: The COVAREP and FACET frameworks are used for feature extraction. These frameworks have a time complexity of *O*(*N* ⋅ *M*), where *N* is the number of samples and *M* is the number of features. The computational cost is primarily associated with the extraction of spectral and visual features. Text Data: The text feature extraction network employs global word vectors and a LSTM network. The time complexity of the LSTM network is *O*(*T* ⋅ *H*), where *T* is the sequence length and *H* is the hidden layer size. The CNN used for feature extraction has a time complexity of *O*(*C* ⋅ *K* ⋅ *H*), where *C* is the number of convolutional filters and *K* is the kernel size.

2) Feature fusion:

The feature fusion module uses tensor Tucker decomposition to integrate features from different modalities. The time complexity of tensor decomposition is *O*(*R*^3^), where *R* is the rank of the tensor. This complexity is manageable due to the efficient implementation of the decomposition algorithm.

3) Federated learning:

The federated learning framework involves decentralized training across edge nodes. The communication complexity is *O*(*T* ⋅ *W*), where *W* is the number of multimodal data samples. The computational complexity at each node is primarily associated with the local model training, which is *O*(*N* ⋅ *M*) for feature extraction and *O*(*T* ⋅ *H*) for the LSTM network.

4) Overall complexity:

The overall time complexity of the proposed approach is *O*(*N* ⋅ *M* + *T* ⋅ *H* + *R*^3^). This complexity is efficient given the distributed nature of the federated learning framework, which allows for parallel processing across multiple edge nodes.

### 3.3. Sub-module design

#### 3.3.1. Feature extraction module.

In this research, we consider three types of multimodal data: audio, visual, and text. Specific sub-networks are employed to extract features from each modality, taking into account their unique characteristics. Raw data from each modality is converted into numerical feature representations (e.g., vectors or tensors) during extraction, enabling compatibility for subsequent tensor-based fusion, rather than maintaining original forms such as raw audio waveforms, image pixels, or text strings. For text, global word vectors (e.g., pre-trained GloVe embeddings) combined with LSTM (or a hybrid LSTM-CNN as in our implementation) capture semantic and contextual dependencies to derive explicit sentiment representations—essential for text-dominant retrieval in TREC2017 and complementary meaning in CMU-MOSI. For audio, COVAREP extracts low-level acoustic descriptors (e.g., pitch, energy, and spectral features) to model prosodic cues indicative of emotional tone, which is particularly valuable for sentiment intensity in CMU-MOSI. For visual data, FACET derives facial action units and expression features to quantify non-verbal emotional signals (e.g., smiles or frowns), enhancing holistic sentiment understanding in CMU-MOSI (not applicable to text-only TREC2017).The adaptive feature extraction mechanism is designed to handle the varying structures of multimodal data, ensuring that the unique characteristics of each modality are effectively captured.

For text-only evaluation, the TREC2017 Precision Medicine Track Scientific Abstracts dataset is employed as a linguistically complex benchmark. The intricate domain-specific terminology and heterogeneous writing structures in TREC2017 serve as a challenging testbed to assess the robustness of the text feature extraction module, the tensor fusion strategy, and the federated aggregation process under high textual variability.

To evaluate the full multimodal capability of the proposed framework, we further incorporate the CMU-MOSI dataset, which contains synchronized text, audio, and visual modalities. For CMU-MOSI, the hybrid LSTM–CNN network is used to extract textual semantic features, COVAREP is applied to obtain acoustic descriptors sampled at 100 Hz, and FACET is utilized to extract facial expression and action-unit–based visual features sampled at 30 Hz. These streams are temporally aligned at the segment level following standard CMU-MOSI preprocessing procedures.

The extracted modality-specific representations from CMU-MOSI are subsequently processed through the proposed high-order tensor Tucker fusion mechanism, enabling the modeling of cross-modal interactions among linguistic cues, prosodic characteristics, and facial behavioral dynamics. This multimodal configuration allows the framework to demonstrate its capacity to handle heterogeneous inputs in real multimodal environments rather than simulated settings.

**Fig 3:**
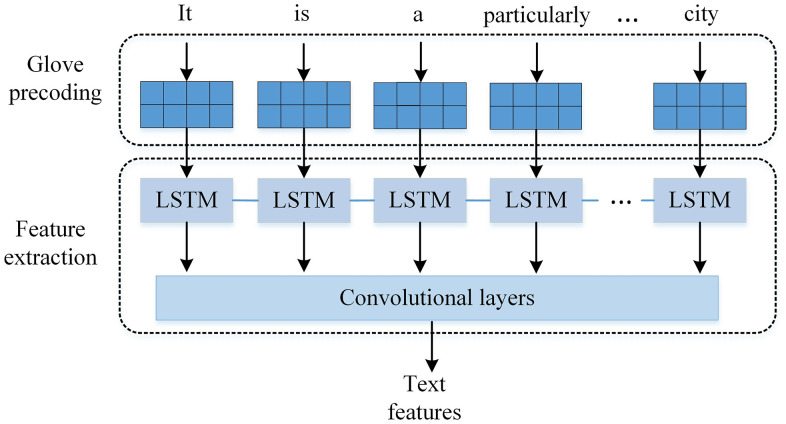
Architecture of the hybrid LSTM-CNN text feature extraction sub-network. The input text passes through an embedding layer, followed by convolutional filters for local feature extraction, max-pooling for dimensionality reduction, and an LSTM layer to capture sequential dependencies. The output is a context-aware text representation used for subsequent multimodal fusion.

In spoken language, there are distinct differences in grammar and expression compared to written text. For instance, spoken language often includes phrases like “I think it’s good..., but I think this method can be improved,” which are less common in written text. Therefore, it is crucial to develop models that can handle the variability of spoken language, focusing on important words and operating effectively in unreliable situations. As illustrated in Fig 3, the proposed text feature extraction network employs global word vectors in the encoding phase to preprocess spoken words.

To address the context-dependent nature of social media text, a hybrid architecture integrating Long Short-Term Memory (LSTM) and Convolutional Neural Network (CNN) components was developed. This architecture is specifically designed to capture both sequential dependencies and local features within text data. The model is structured as follows.

(1) Embedding Layer: The input text is first transformed into dense word embeddings using pre-trained global word vectors (e.g., GloVe or Word2Vec). These embeddings, typically of 300 dimensions, capture semantic relationships between words and serve as the input to the subsequent layers.
(2) CNN Layer: A convolutional neural network with multiple filter sizes (e.g., 3, 4, 5) is applied to the embedded text to extract local features. Each filter slides over the text to capture n-gram features, producing feature maps that are processed through a ReLU activation function to introduce nonlinearity.
(3) Max-Pooling Layer: The feature maps from the CNN layer are subjected to max-pooling, which reduces dimensionality while retaining the most prominent features from each filter, thus enhancing computational efficiency.
(4) LSTM Layer: The pooled features are fed into an LSTM network with 128 hidden units. This layer captures the sequential dependencies and contextual information in the text, adapting to the variable length and structure of spoken language. A dropout rate of 0.5 is applied to prevent overfitting during training, as this is a commonly used value in deep learning models [27] and was found to perform optimally through cross-validation on the training dataset.
(5) Output Layer: The LSTM output is passed through a fully connected layer with a softmax or linear activation (depending on the downstream task) to produce the final text feature representation.

This hybrid LSTM-CNN architecture effectively addresses the challenges posed by context-dependent social media text by leveraging the CNN’s capability to extract fine-grained local features and the LSTM’s strength in modeling long-term dependencies. Hyperparameters, including filter sizes, the number of filters (e.g., 128 per size), and LSTM units, were optimized via cross-validation on the training dataset to ensure peak performance.

The proposed framework incorporates an adaptive feature extraction mechanism to handle the heterogeneous nature of multimodal data in social media. This mechanism dynamically adjusts the feature extraction process based on the structural characteristics of the input data. Specifically, although we employ established frameworks such as LSTM-CNN for text, COVAREP for audio, and FACET for visual signals, we have tailored their integration for the noisy, heterogeneous, and dynamic context of social media data. For instance, our system includes an adaptive selection and weighting mechanism that prioritizes the most informative features based on modality-specific reliability (e.g., giving more weight to text when visual or audio data are missing or degraded). This design ensures that the feature extraction modules are not simply reused, but are adapted to cope with the distinct challenges posed by real-world social media environments, where data quality and structure can vary widely across users and devices. For example, in text data, the LSTM-CNN model evaluates the sequence length and contextual complexity of the input and adjusts its parameters (e.g., kernel size, window size) to optimize feature extraction. The CNN network extracts fine-grained and localized features from the textual information through convolutional kernels applied in the convolutional layer. The LSTM network in this sub-network can dynamically adjust to the variability of spoken language, ensuring robust feature extraction.

To further reduce computation overhead on edge devices, we employ lightweight feature extraction techniques. For audio and visual data, the COVAREP and FACET frameworks are optimized for low computational complexity. For text data, the combination of global word vectors and LSTM ensures efficient feature extraction. Additionally, tensor decomposition techniques are used to compress the model parameters, reducing the computational and storage requirements. This makes the model feasible for deployment on resource-constrained edge devices.

#### 3.3.2. Feature fusion module.

**Fig 4:**
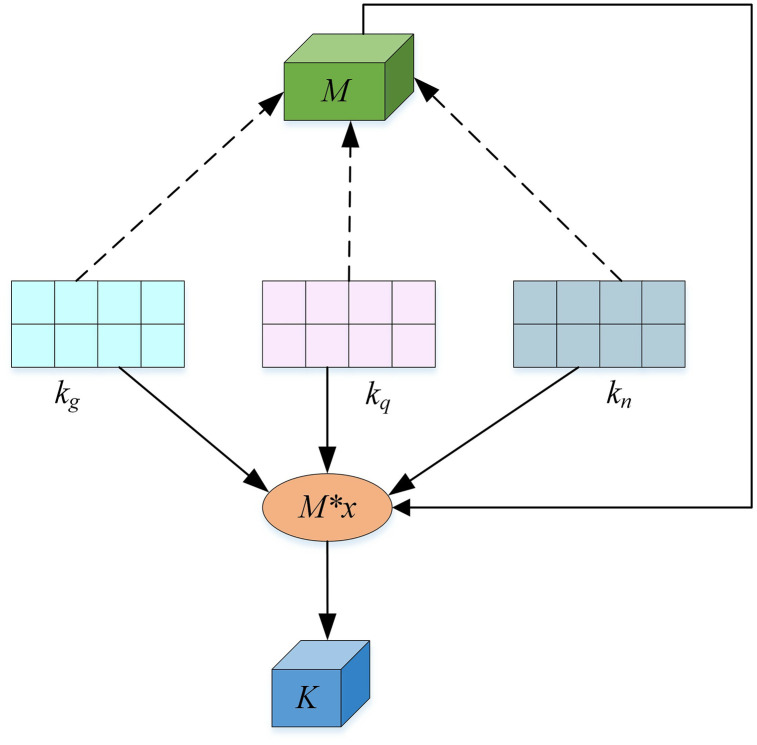
Multimodal data fusion based on Tucker decomposition. $k_{\text{g}},k_{\text{q}}$, and $k_{\text{n}}$ denote the audio, visual, and text feature matrices, respectively, while *M* represents the learnable core tensor. The symbol “×” indicates mode-*n* tensor–matrix multiplication, and *M* × *x* illustrates the intermediate fusion state during successive mode-wise multiplications. The final fused multimodal representation is denoted as *K*.

We consider the audio data features $k_{\text{g}}=\left(\overset{\text{1}}{\underset{\text{g}}{k}},\overset{\text{2}}{\underset{\text{g}}{k}},\cdots ,\overset{v}{\underset{\text{g}}{k}}\right)$, visual data features $k_{\text{q}}=\left(\overset{\text{1}}{\underset{\text{q}}{k}},\overset{\text{2}}{\underset{\text{q}}{k}},\cdots ,\overset{u}{\underset{\text{q}}{k}}\right)$, and text data features $k_{n}=(\overset{\text{1}}{\underset{n}{k}},\overset{\text{2}}{\underset{n}{k}},\cdots ,\;\;\overset{w}{\underset{n}{k}})$. After passing through the feature fusion module, the output features are denoted as *K*. As depicted in Fig 4, our approach incorporates a higher-order tensor *M* that represents the multimodal data feature space. To address the challenge of underutilized intermodal correlations, the proposed method employs tensor Tucker decomposition. This technique decomposes a high-order tensor into a core tensor, which captures the complex interactions between modalities, and factor matrices, which encapsulate modality-specific features. To further support this, we iteratively perform modulo multiplication between the memory unit and the multimodal features along different orders, preserving their interdependencies while enhancing the fused representation. This process demonstrates that our fusion mechanism not only incorporates multimodal data into a memory cell but also enables robust feature fusion operations, as evidenced by its ability to produce a comprehensive tensor *K*. By performing modulo multiplication between the memory unit and the multimodal features along different orders, we iteratively integrate the features while preserving their correlations. This process ensures that the fused tensor *K* effectively combines the characteristics of all modalities, utilizing their interdependencies to enhance the overall representation.

Let’s use Fig 4 as a reference. Suppose the multimodal data features to be processed are represented as $k_{\text{g}},k_{\text{q}},k_{\text{n}}$. In this case, the memory cell *M* is a third-order tensor. In the proposed multimodal data feature fusion approach, we perform a modulo multiplication between the multimodal data features and the corresponding feature space of the memory cell. This allows us to obtain a memory cell that incorporates the multimodal data features, enabling further feature fusion operations. The fusion operation consists of three stages. First, we perform a modulo multiplication between the memory unit *M* and the multimodal data feature $k_{g}$ along the first order. This operation results in a new memory unit $M^{\left(\text{1}\right)}$ with the features of $k_{g}$. Next, we perform a modulo multiplication between the memory unit $M^{\left(\text{1}\right)}$ and the multimodal data feature $k_{q}$ along the second order. As a result, we obtain the memory unit $M^{\left(\text{2}\right)}$ with the features of $k_{g}$ and $k_{q}$. Finally, we perform a modulo multiplication between the memory cell $M^{\left(\text{2}\right)}$ and the multimodal data feature $k_{\text{n}}$ along the third order. This step produces the fusion tensor *K*, which encompasses the characteristics of all three multimodal data $k_{\text{n}}$. The specific process can be expressed as follows:


$$K=\left(\left(M\times_{\text{1}}k_{g}\right)\times_{\text{2}}k_{q}\right)\times_{\text{3}}k_{n}$$ (1)

where $M\in R^{R_{\text{1}}\times R_{\text{2}}\times R_{\text{3}}}$, $k_{g}\in R^{U\times R_{\text{1}}}$,$\;k_{n}\in R^{Z}\times R_{\text{3}}$.

The fusion module receives temporally aligned modality-specific feature vectors from the extraction process (per CMU-MOSI standards): text yields a 128-dimensional vector ($d_{t}$= 128, from LSTM after 300D GloVe); audio produces 74D ($d_{a}$ = 74, with MFCCs, pitch, glottal params); visual gives 35D ($d_{v}$ = 35, including action units, expressions, landmarks). These form a third-order tensor $X\in R^{d_{k}\times d_{a}\times d_{v}}$ for fusion. Dimensionality reduction uses Tucker decomposition:$X\approx G\times_{\text{1}}U_{t}\times_{\text{2}}U_{a}\times \;\text{3}U_{v}$, where core $G\in R^{R\times R\times R}$ captures interactions, and factors $U_{t}\in R^{d_{t}\times R}$, $U_{a}\in R^{d_{a}\times R}$, $U_{v}\in R^{d_{v}\times R}$ project to low-rank subspace $\left(R\ll min\left(d_{t},d_{a},d_{v}\right),\text{ e}.\text{g}.,\;R=\text{2}\text{0}\right)$. This cuts parameters from $d_{t}\cdot d_{a}\cdot d_{v}\text{ to }R^{\text{3}}+R\left(d_{t}+d_{a}+d_{v}\right)$, enabling efficient compression with preserved correlations.

Where *U* is the factor matrices obtained through Tucker decomposition. The symbols *g*, *q*, *n* denote modality indices corresponding to audio, visual, and text modalities, respectively. The superscripts *v*, *u*, *w* indicate the dimensionality of the feature vectors produced by the corresponding modality-specific feature extractors. Specifically, $k_{g}\in R^{v},k_{q}\in R^{u}$ and $k_{n}\in R^{w}$, where $v=d_{a},u=d_{v}$, and $w=d_{t}$.

#### 3.3.3. Feature decision module.

The feature decision module is a task-adaptive prediction layer that generates final outputs (e.g., class probabilities or continuous values) from the fused tensor *K*. In the decision module, we employ a conventional fully connected layer to make predictions. The aim of the feature decision fusion module is to integrate the fused multimodal representations (from tensor Tucker decomposition) into a cohesive output for downstream tasks, such as sentiment classification or public opinion monitoring, enabling end-to-end prediction while maintaining the privacy benefits of the federated setup. The conventional fully connected layer helps in making decisions by learning a non-linear mapping from the high-dimensional fused feature space to the target output space (e.g., class probabilities via softmax for classification), effectively combining intermodal correlations captured earlier to produce accurate, task-specific predictions.

The choice of loss function depends on the specific task:

For regression tasks: We use the *L*1 loss function to measure the error between the predicted values and the true values. This is appropriate for regression tasks, where the goal is to predict a continuous value. The L1 loss function is defined as shown in Equation 2:


$$L\text{1}Loss=\frac{\text{1}}{N}\sum_{i=\text{1}}^{N}\;\left|y_{i}-{\hat{y}}_{i}\right|$$ (2)

where $y_{i}$ is the ground-truth sentiment score (true value) and ${\hat{y}}_{i}$ is the predicted sentiment score (predicted value) for the *i*-th sample in the multimodal dataset (e.g., a video segment comprising aligned text, audio, and visual features from benchmarks like CMU-MOSI). The indexing *i* refers to individual data samples across the training or evaluation set, enabling computation of mean squared error for regression-based sentiment intensity prediction.

For classification tasks: The *L*1 loss function is not typically used for classification tasks. Instead, we use the cross-entropy loss function, which is more suitable for measuring the error between predicted probabilities and true labels. The cross-entropy loss function is defined as:


$$\text{Cross Entropy Loss }=-\frac{\text{1}}{N}\sum_{i=\text{1}}^{N}\;\sum_{c=\text{1}}^{C}\;\overset{c}{\underset{i}{y}}log\left(\overset{c}{\underset{i}{\hat{y}}}\right)$$ (3)

where $\overset{c}{\underset{i}{y}}$ is the true label for class *c* and $\overset{c}{\underset{i}{\hat{y}}}$ is the predicted probability for class *c*, with class *c* representing a sentiment or opinion category (e.g., positive, negative, or neutral) derived from the fused multimodal model integrating text, audio, and visual features, rather than from any single modality.

### 3.4. Multimodal data fusion based on federated learning

Let’s denote the number of edge nodes as *T*. Additionally, let’s assume that all edge nodes collectively gather a total sample size of *W* multimodal data samples. During the initialization phase, the shared model *A* can be represented as follows:


A=⟨F,X,C⟩ (4)

where ⟨·⟩ denotes the stitching operation, the angle brackets ⟨ ⋅ , ⋅ , ⋅ ⟩ denote the federated aggregation operation (e.g., weighted averaging of local updates), combining local model parameters *F*, the current global model *X*, and core tensor components *C* to produce the updated global parameter *A*. More specifically, within the feature extraction module *F*, corresponding feature extraction sub-networks $F_{\text{1}},F_{\text{2}},\cdots ,F_{W}$ are designed for each of the *W* multimodal data, which can be expressed as


$$F=\left\langle F_{\text{1}},F_{\text{2}},\cdots ,F_{W}\right\rangle$$ (5)

where $F_{x}$ denotes the feature extraction sub-network for the *x*-th multimodal data. Note that *W* indexes the distributed samples/nodes in the federated setting and is unrelated to fused features from individual modalities. After training, the parameters of this tensor are expanded along the *x*-th mode to reflect the spatial dimensional characteristics of the *x*-th multimodal data.

By Equation (1), it can be seen that when $R_{\text{1}}=U,R_{\text{2}}=Y,R_{\text{3}}=Z$, the size of the fused tensor is the same as the size of the memory unit *M*, which are $K\in R^{R_{\text{1}}\times R_{\text{2}}\times R_{\text{3}}}$.

**Fig 5:**
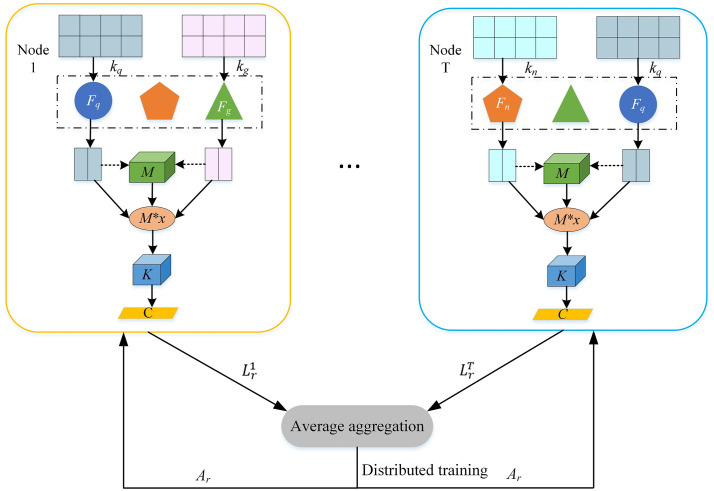
Federated learning process for multimodal data fusion. Each node performs local feature extraction and fusion, followed by uploading model updates to the central server. The server aggregates these updates to refine the global model, which is then redistributed to the nodes for the next training round, ensuring privacy preservation and collaborative learning.


As shown in Fig 5, the adaptive selection mechanism of the feature extraction module is described in more detail in this section. Based on the variety of multimodal data types present at the nodes, the feature fusion phase can be categorized into two components. The procedure can be described as follows.

$$\begin{array}{c} M^{\left(\text{1}\right)}=X\left(f_{g};M\right),\\ K=X\left(f_{q};M^{\left(\text{1}\right)}|M\right). \end{array}$$ (6)

In the fusion process, $M^{\left(\text{1}\right)}|M$ represents the features of $f_{q}$. The model initially utilizes the memory unit to store the $f_{g}$ features, resulting in a model with $f_{g}$ features. This stored information serves as prior knowledge when fusing the $f_{q}$ features. Thus, throughout the model training phase, the memory unit acquires the spatial characteristics of each multimodal data and discriminates potential connections between different multimodal data types.

The training mechanism on node *T* follows a similar process as node 1. The described procedure can be represented as following:


$$\overset{z}{\underset{r}{L}}=\overset{z}{\underset{r-\text{1}}{L}}-\eta \overset{z}{\underset{r}{a}}$$ (7)

where $\overset{z}{\underset{r}{L}}$ is the multimodal data collected using local acquisition on node *z* in the *r*-th round of global iterations. Here, r indexes the global communication rounds, and z indexes the individual edge nodes. These indices are standard in federated learning to denote distributed training dynamics and are distinct from the modality-specific indexing used in earlier discussions of feature fusion and decision modules. The local model obtained by the gradient descent algorithm with learning rate η, and $\overset{z}{\underset{r}{a}}$ is the corresponding gradient. In the model aggregation phase, feature extraction sub-networks from edge nodes are combined to create a shared model. This is achieved by selecting the best feature extractor and applying an average aggregation algorithm. The procedure can be represented as follows


$$F^{*}=\left\langle \sum_{z=\text{1}}^{T}\;\frac{\overset{z}{\underset{\text{1}}{t}}}{t_{\text{1}}}\times \overset{z}{\underset{\text{1}}{F}},\sum_{z=\text{1}}^{T}\;\frac{\overset{z}{\underset{\text{2}}{t}}}{t_{\text{2}}}\times \overset{z}{\underset{\text{2}}{F}},\cdots ,\sum_{z=\text{1}}^{T}\;\frac{\overset{z}{\underset{W}{t}}}{t_{W}}\times \overset{z}{\underset{W}{F}}\right\rangle$$ (8)

Thus the global shared model after the *r*-th round of updates is


$$A_{r}=\left\langle F^{*},X^{*},C^{*}\right\rangle$$ (9)

It can also be expressed as follows.


$$A_{r}=\left\langle F^{*},X^{*},C^{*}\right\rangle =\left\langle F^{*},\sum_{z=\text{1}}^{T}\;\frac{w^{z}}{w}\times X^{z},\sum_{z=\text{1}}^{T}\;\frac{w^{z}}{w}\times C^{z}\right\rangle$$ (10)

where $A_{r}$ represents the shared model with global features achieved through the aggregation process using the joint averaging algorithm. $w^{z}=\sum_{x=\text{1}}^{w}\;\overset{z}{\underset{x}{t}}$ and $w=\sum_{x=\text{1}}^{w}\;t_{x}$ are the total count of multimodal samples and the cumulative count of multimodal samples, respectively.

The adaptive selection mechanism of the feature extraction module ensures that the proposed framework can handle diverse data types from heterogeneous devices. This flexibility is crucial for large-scale deployments, as it allows the system to dynamically adjust to the varying data formats and computational capabilities of different devices. Unlike conventional federated learning approaches that apply uniform averaging across all edge nodes update, our framework introduces a modality-aware adaptive aggregation mechanism. Specifically, we dynamically adjust the weight assigned to each edge node’s update during the aggregation phase based on both (a) the quality of the modality-specific data (e.g., noisy or incomplete modalities) and (b) the local performance contribution (measured through cross-validation). This design ensures that the global model prioritizes high-quality, informative updates over noisy or less relevant contributions, which is especially important in the social media context where edge nodes often operate under heterogeneous and non-IID data distributions. This adaptation distinguishes our approach from prior generic federated learning methods, improving both robustness and final model performance, as demonstrated in the experimental results. Furthermore, the federated learning approach reduces the communication overhead by only transmitting model parameters instead of raw data, making it feasible to deploy the system across a wide range of devices with limited bandwidth and processing power.

## 4. Result analysis and discussion

The experimental evaluation comprises two complementary datasets. The TREC2017 Precision Medicine Track Scientific Abstracts dataset (text-only) is used as a controlled benchmark to validate the behavior of the proposed feature extraction, tensor fusion, and federated aggregation mechanisms in linguistically complex retrieval tasks. To directly assess multimodal fusion performance, we additionally evaluate the full pipeline on the CMU-MOSI multimodal sentiment benchmark (text + audio + visual). The two datasets serve different purposes: TREC2017 tests the method’s performance under text-dominant conditions, while CMU-MOSI evaluates the ability of the high-order tensor fusion to capture intermodal correlations in real multimodal data. The experiments in this paper are conducted in a simulated or centralized environment to evaluate the proposed framework’s performance. A full deployment on distributed edge nodes devices with realistic communication constraints is left as future work.

To ensure experimental transparency and reproducibility, we provide the following implementation details. The hybrid LSTM–CNN network for text feature extraction consisted of one convolutional layer with filter sizes of 3, 4, and 5, each with 128 filters, followed by a max-pooling layer and an LSTM layer with 128 hidden units and a dropout rate of 0.5. The fully connected output layer used a softmax activation for classification. The tensor Tucker decomposition rank R was empirically set to 20, balancing model complexity and computational efficiency. The model was trained for 100 epochs with a batch size of 64, using the Adam optimizer (learning rate = 1 × 10 ^−^ ³, β₁ = 0.9, β₂ = 0.999). In the federated learning setup, each edge node performed 5 local epochs per communication round, and the global model was updated via weighted averaging across nodes.

All experiments were conducted on a workstation equipped with an NVIDIA RTX 3090 GPU (24 GB VRAM), an AMD Ryzen 9 5950X CPU, and 64 GB RAM, running Ubuntu 22.04 LTS with PyTorch 2.2.1. These configurations ensure reproducibility and provide a realistic simulation of edge-device-based federated environments.

After feature fusion, the queries in each group of member systems are divided into odd and even groups using bifold cross-validation. First, the even-numbered groups in the nodes are selected using a sequential forward algorithm using a greedy strategy to select the member system groups, after which they are fused and tested on the odd-numbered queries in the corresponding member system groups using CombSUM as the computational evaluation metric, and then tested in reverse. CombSUM, CombMNZ, and MR are used as the post-selection fusion methods in the experiments, and the MAP value is used as the fusion performance evaluation index of the algorithm. The experiments are divided into two parts.

### 4.1. Performance control on small-scale data sets

To evaluate the proposed algorithm, we compared it against several established and recent state-of-the-art methods. These include: Lu et al.‘s HOGA-SVM [28], Boughanmi et al.’s multimedia data fusion [29], Ghazal’s machine learning architecture [30], as well as the recent adaptive meta-learning framework by Yang et al. [22] and the hierarchical attention mechanism by Dui et al. [23]. For the experiment, 50 member systems with better MAP values were selected. The member systems are divided into 10 nodes, and 2–10 member systems are selected in turn. All-List denotes the results of the fusion of all member systems. The experimental results are shown in Table 1.

**Table 1:** MAP values of different algorithms.

Algorithm	2	3	4	5	6	7	8	9	10
Lu et al. [28]	0.759	0.775	0.784	0.791	0.803	0.788	0.781	0.777	0.767
Boughanmi et al. [29]	0.818	0.827	0.835	0.842	0.854	0.843	0.834	0.826	0.812
Ghazal [30]	0.841	0.852	0.863	0.884	0.896	0.877	0.855	0.848	0.845
Yang et al. [22]	0.825	0.838	0.849	0.861	0.872	0.859	0.847	0.835	0.822
Dui et al. [23]	0.831	0.845	0.858	0.869	0.881	0.866	0.852	0.841	0.829
Proposed	0.863	0.874	0.892	0.916	0.935	0.921	0.918	0.887	0.876

The analysis of Table 1 reveals that the performance of all algorithms follows a trend of increasing and then decreasing as the number of selected member systems increases, with optimal performance observed at approximately 6 systems. Notably, the proposed algorithm consistently outperforms all baseline methods, including the recent state-of-the-art approaches [22,23]. For instance, at the optimal point, our method achieves a MAP of 0.935, which represents a significant improvement over Yang et al. [22] (0.872) and Dui et al. [23] (0.881). This performance gain can be attributed to our method’s ability to more effectively capture complex intermodal correlations through tensor-based fusion, a capability that is less pronounced in the attention or meta-learning mechanisms of the comparator methods.

### 4.2. Performance experiments on large data sets

**Fig 6:**
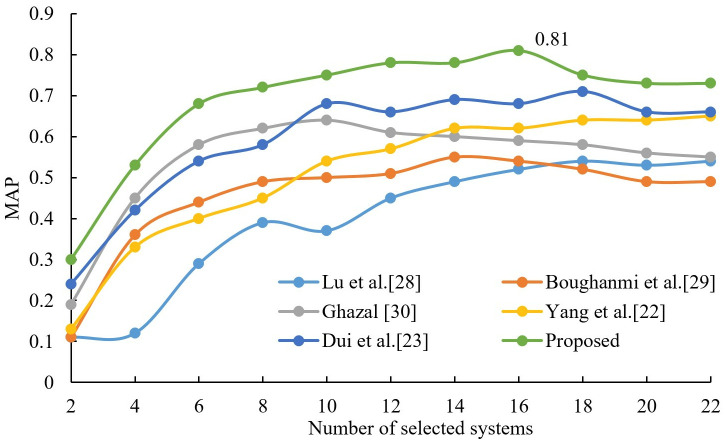
Comparison results of different algorithms on CombSUM.

**Fig 7:**
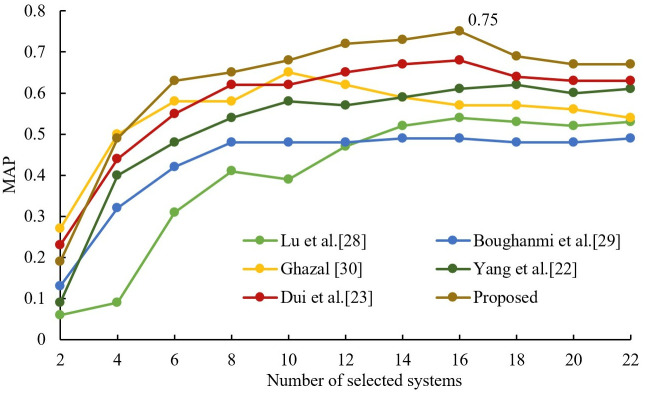
Comparison results of different algorithms on CombMNZ.

**Fig 8:**
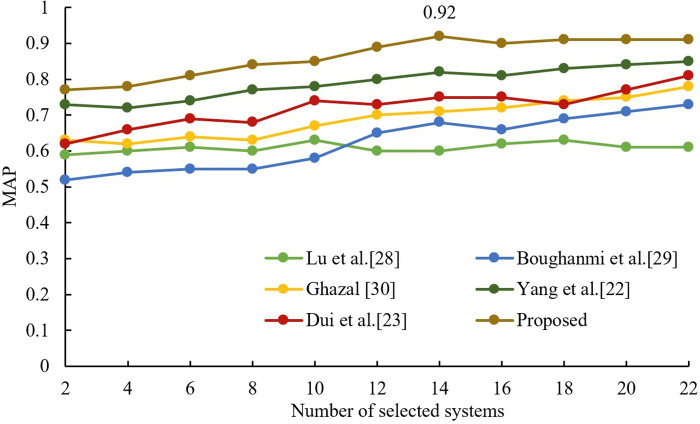
Comparison results of different algorithms on MR.

In order to illustrate the performance of this algorithm on large data sets, this section uses a data set with 108 member systems (i.e., participating retrieval systems’ submitted runs, as described in Section 4.1) to test. After the experimental test, the dataset is divided into 21 node numbers, so different groups (from 2 to 21 groups) are selected for the fusion experiments, and three other comparison algorithms are introduced. Lu et al.’s HOGA-SVM algorithm [28] used the genetic algorithm as a membership system. Boughanmi and Ansari’s multimedia data fusion approach [29] sequentially selected the member systems with larger MAP values according to the MAP table to participate in the fusion. On the other hand, Ghazal’s data fusion-based architecture [30] selects the member system with the largest MAP value in each node, in turn, to participate in the fusion after the feature fusion is completed. These four algorithms were applied to the experimental dataset and CombSUM, CombMNZ, and MR were used as the fusion methods for the member system groups, respectively. The results are shown in Figs 6–8. From observing Figs 6–8, it is evident that the fusion performance of all proposed selection algorithms improves gradually with an increase in the number of selected member systems. The best performance is the proposed algorithm, and Ghazal’s data fusion-based architecture [30] is the second best. When employing CombSUM, CombMNZ, and MR for fusion, the proposed algorithm achieves peak MAP values of 0.81, 0.75, and 0.92 at 16, 16, and 14 member systems, respectively.

### 4.3. Evaluation on multimodal dataset

To further evaluate the applicability of the proposed method in genuine multimodal scenarios, additional experiments were conducted on the CMU-MOSI multimodal sentiment analysis dataset, which contains synchronized text, audio, and visual streams. The CMU-MOSI dataset is a widely used benchmark for multimodal sentiment analysis and contains synchronized text, audio, and visual streams. It consists of opinion-expressing YouTube videos with fine-grained sentiment intensity annotations, making it highly suitable for our research as it mirrors real-world social media content and enables rigorous assessment of high-order tensor fusion in capturing intermodal correlations for sentiment analysis tasks. Each video segment is annotated with sentiment intensity scores, providing a natural setting for multimodal fusion evaluation.

For benchmarking, we include several recent multimodal learning methods already cited in this study: Yang et al. [22], Dui et al. [23], and Chen et al. [24]. Performance was evaluated using accuracy and MAP, which are standard metrics for multimodal sentiment recognition. The results are presented in Table 2.

**Table 2:** Multimodal performance comparison on CMU-MOSI.

Method	Text modality	Audio modality	Visual modality	Accuracy	MAP
Yang et al. [22]	✓	✓	✗	0.782	0.764
Dui et al. [23]	✓	✓	✓	0.801	0.779
Chen et al. [24]	✓	✓	✓	0.828	0.803
Proposed (Ours)	✓	✓	✓	0.856	0.832

As shown in Table 2, the proposed method achieves the highest accuracy (0.856) and MAP (0.832) among all baselines on CMU-MOSI. The gains over Chen et al. [24] demonstrate the effectiveness of the tensor-based fusion mechanism in modeling high-order cross-modal dependencies, while the federated training structure contributes to more robust feature generalization across distributed nodes. These results confirm that the proposed framework is capable of handling real multimodal data and that the high-order tensor fusion substantially enhances intermodal correlation modeling.

### 4.4. Alllist comparison

**Fig 9:**
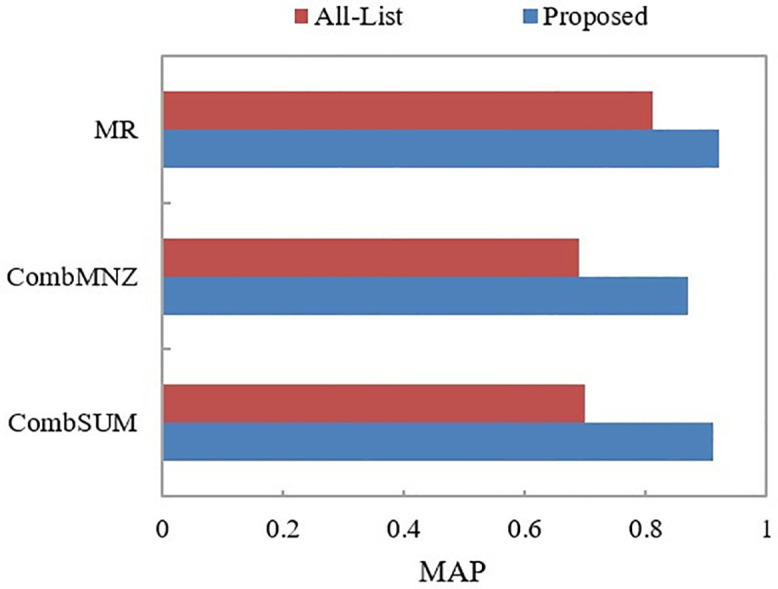
Performance comparison of the proposed method and all-member system fusion.

The proposed approach is compared to the All-List method, as illustrated in Fig 9. By examining Fig 9, it becomes evident that the fusion performance of the proposed approach, which combines member result lists, surpasses that of fusing all member result lists. Moreover, the proposed method demonstrates a significant reduction in the number of required member systems (i.e., participating retrieval systems from TREC2017), leading to a notable decrease in time complexity and an enhancement in fusion efficiency.

### 4.5. Ablation study

To further evaluate the impact of each component in our proposed method, we conducted ablation experiments by removing specific components and observing the performance changes. The results are presented in Table 3.

**Table 3:** Ablation study results.

Model	MAP value
Full model(Ours)	0.935
Without Feature extraction	0.854
Without Feature fusion	0.821
Without Federated learning	0.789

From Table 3, it can be seen that removing the customized feature extraction modules results in a MAP value of 0.854, a drop of 8.7% compared to the full model. This shows the importance of feature extraction in capturing modality-specific characteristics. Removing the tensor-based feature fusion mechanism results in a MAP value of 0.821, a drop of 12.2% compared to the full model. This highlights the effectiveness of tensor-based fusion in integrating multimodal features. Removing the federated learning strategy results in a MAP value of 0.789, a drop of 15.6% compared to the full model. This underscores the critical role of federated learning in preserving privacy and enhancing model performance. The ablation study demonstrates that each component of our proposed method contributes significantly to the overall performance. The full model achieves the highest MAP value, indicating that the integration of all components is essential for optimal performance.

Our experimental results demonstrate that the proposed method achieves higher MAP values compared to existing approaches, which can be attributed to the effective utilization of intermodal correlations through tensor-based fusion. By capturing the complex relationships between different modalities, our approach outperforms traditional methods that often fail to fully exploit these correlations. This improvement in MAP values indicates that our method is better at retrieving relevant information, which is crucial for applications like sentiment analysis and content recommendation in social media contexts.

## 5. Conclusion

This study addresses the challenge of privacy-aware multimodal data fusion on edge devices, where data sharing is restricted by regulations. By employing tensor decomposition theory, we developed an advanced memory unit that encapsulates the spatial attributes of multimodal data, enabling efficient data fusion while supporting user privacy through local data retention. Our method enhances learning capabilities without complicating the model architecture. In particular, our high-order tensor-based fusion overcomes the complexities of integrating heterogeneous modalities (audio, visual, and text) by leveraging Tucker decomposition to model intricate intermodal correlations, reducing parameter redundancy and handling varying data structures more effectively than traditional concatenation or independent processing. Furthermore, data from each modality—with dimensions such as 128 for text, 74 for audio, and 35 for visual—are first extracted into numerical vectors via modality-specific sub-networks and projected to a common latent space, enabling seamless tensor construction and fusion for downstream tasks.

To summarize how the proposed method addresses the difficulties of fusing different modalities: (i) raw data in diverse forms (waveforms, pixels, text) are first converted into fixed-dimensional numerical vectors by modality-specific extractors (LSTM–CNN for text, COVAREP for audio, FACET for visual), which resolves the heterogeneity of formats and dimensions; (ii) these vectors are then organised into a third-order tensor and fused via Tucker decomposition and mode-n products with a learnable core tensor, so that intermodal correlations are captured without requiring identical dimensions across modalities; (iii) the fused tensor is fed into the feature decision module (fully connected layer) for task-specific prediction. Thus, differing dimensions from each modality are unified through extraction and then fused in a single tensor representation before being passed to the decision stage. Experimental results demonstrate superior performance compared to existing data fusion techniques, making it a promising approach for applications requiring privacy-aware and efficient data handling. The present work does not yet provide a formal privacy analysis, but rather focuses on the architectural integration of tensor fusion within a federated paradigm.

Future work should include comprehensive privacy evaluation metrics, such as differential privacy analysis and secure aggregation protocols, to provide stronger privacy guarantees in federated learning scenarios. When applying such models to user-generated content, ethical considerations (e.g., consent, anonymisation, potential harm) and compliance with relevant regulatory frameworks are important; federated architectures can support better data governance by keeping data on-device.
